# Knowledge of Chronic Kidney Disease among the General Population: A Questionnaire Survey in Hokkaido Prefecture, Japan

**DOI:** 10.3390/jpm12111837

**Published:** 2022-11-03

**Authors:** Naoki Nakagawa, Saori Nishio

**Affiliations:** 1 Division of Cardiology, Nephrology, Pulmonology and Neurology, Department of Internal Medicine, Asahikawa Medical University, Asahikawa 078-8510, Japan; 2 Division of Rheumatology, Endocrinology and Nephrology, Graduate School of Medicine, Hokkaido University, Sapporo 060-8648, Japan

**Keywords:** knowledge, chronic kidney disease, perception, questionnaire, Japan

## Abstract

Public education programs about chronic kidney disease (CKD) are important activities worldwide. The present study investigated the knowledge of CKD in the general population of 58 out of 179 cities or towns in Hokkaido between 1 April and 30 September 2019. A total of 15,012 respondents who underwent specific health checkups at these centers answered the questionnaire. In response to a questionnaire item asking about the respondent’s familiarity with the term “CKD”, only 6% of the respondents answered “know it well” and 13% answered “heard of it”. In contrast, in response to a questionnaire item asking about the respondent’s familiarity with “chronic kidney disease”, 31% answered “know it well” and 33% answered “heard of it”. The leading avenue by which the respondents learned about CKD was television, followed by newspapers, magazines, and a family doctor or nurse. The leading component that the respondents considered essential for the diagnosis of CKD was proteinuria. These results indicated that the knowledge of CKD in Hokkaido prefecture is still inadequate. Many people did not appear to realize that the term “CKD” represents “chronic kidney disease”. Further continuous public education efforts are required to enlighten people about CKD.

## 1. Introduction

In Japan, the number of patients from end-stage kidney failure to chronic dialysis therapy (hemodialysis and peritoneal dialysis) is increasing every year [1,2]. Furthermore, the number of dialysis patients per capita is the second-largest in the world. The total annual medical cost related to dialysis is about JPY two trillion and the increase in the number of chronic maintenance dialysis patients is a major economic health-care problem. Chronic glomerulonephritis used to be the most-common cause of dialysis induction, until diabetic nephropathy began to lead from 1998 and nephrosclerosis associated with hypertension has been increasing every year [3]. It has been reported that dialysis patients are at high risk of developing acute myocardial infarction and cerebrovascular disease and have a high mortality rate after development of these diseases [4,5]. The relative risk of cardiovascular death increases as urinary protein increases from trace albuminuria to overt albuminuria. Furthermore, the relative risk increases with a glomerular filtration rate (GFR) < 60 mL/min/1.73 m^2^ and increases as kidney function declines [6,7].

Against this background, the importance of diagnosis and treatment of kidney impairment has been recognized. The concept of chronic kidney disease (CKD) was proposed by the American Kidney Foundation in 2002 [8], which defined CKD as (1) laboratory findings indicating the presence of kidney disease, such as proteinuria, (2) decreased kidney function (estimated GFR (eGFR) < 60 mL/min/1.73 m^2^), or both, persisting for at least three months. CKD is not only a prelude to end-stage kidney failure, but also a risk factor for complications of cardiovascular disease (CVD), which is associated with atherosclerosis [9]. Therefore, CKD control is an important issue in maintaining the health of the population.

In order to further promote measures against kidney diseases in Japan, the first “Kidney Disease Control Commission Meeting” was held in December 2017 over the course of four meetings in the Ministry of Health, Labour and Welfare (MHLW) of Japan [10]. This commission aims to prevent CKD exacerbation through early detection and diagnosis based on a few subjective symptoms, to implement and continue appropriate high-quality treatment and to maintain and improve the quality of life (QOL) of patients with CKD, including those receiving dialysis and kidney transplant. The activities identified for future implementation are organized into each of the following five categories: “raising public awareness,” “improving regional health care provisions”, “improving the level of medical care”, “developing human resources”, and “promoting research and development”. In addition, key performance indicators (KPIs) are set to reduce the number of new dialysis patients to ≤35,000 by 2028 [10]. One of the most important efforts is to educate the general public about CKD [11,12]. However, it is not clear to what extent the terms “CKD” and “chronic kidney disease” are well known to the general public in Hokkaido. Therefore, we conducted a survey to understand the current level of knowledge of CKD in Hokkaido.

## 2. Materials and Methods

This study included 15,122 participants who agreed to answer a questionnaire among those who received specific health checkups in 58 out of 179 cities or towns in Hokkaido between 1 April and 30 September 2019. The questionnaires (Supplementary Table S1) were hand delivered to the participants and collected after they responded to them.

This study was approved by the Asahikawa Medical University Institutional Review Board (No. 22036) and was conducted in accordance with the Declaration of Helsinki.

All collected questionnaires were sent to Asahikawa Medical University for data analysis. Statistical analysis was performed using the Kruskal–Wallis test and Bonferroni correction as a multiple-comparison test using IBM SPSS v.26.0 (SPSS, Chicago, IL, USA). A *p*-value < 0.05 was considered statistically significant.

## 3. Results

### 3.1. Participants and Their Characteristics

**Figure 1:**
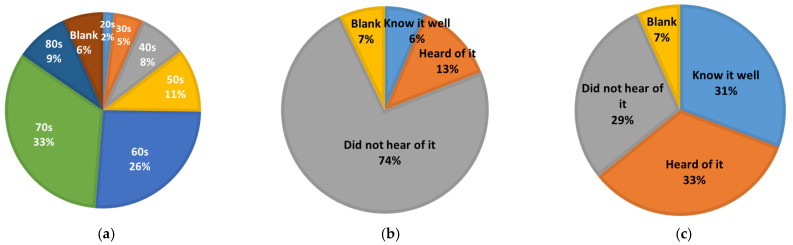
Participants and perception of CKD in the general Hokkaido population. (**a**) Age distribution of participants; (**b**) perception of “CKD”; (**c**) perception of “chronic kidney disease”.

Of the 15,012 respondents, 5138 (34%) were male, 7767 (52%) were female, and 2024 (14%) were non-responsive. By age, 33% and 26% of the respondents were in their 70s and 60s, respectively, followed by 11% in their 50s and 9% in their 80s (Figure 1a).

### 3.2. Perception of CKD in the General Hokkaido Population

Regarding knowledge of CKD and chronic kidney disease, 6% of the total respondents were familiar with “CKD”, 13% had “heard of it”, and 74% had “never heard of it” (Figure 1b). Conversely, 31% of the respondents were familiar with “chronic kidney disease”, 33% had “heard of it”, and 29% had “never heard of it”. The recognition level of “CKD” and “chronic kidney disease” was clearly different (Figure 1c). The percentage of respondents who did not respond to either question was 7%. 

**Figure 2:**
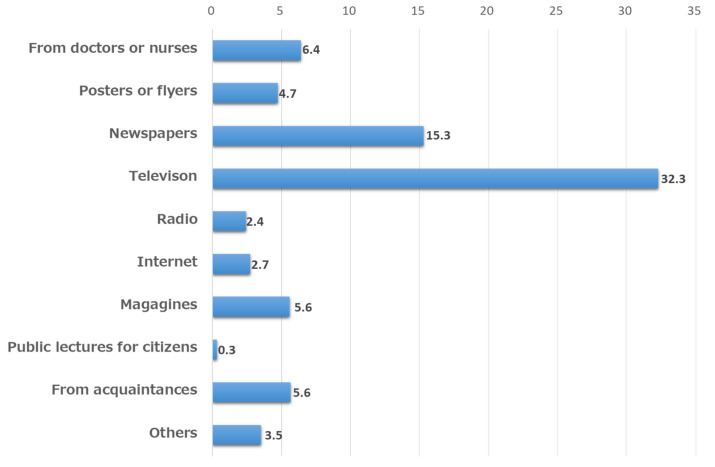
Opportunity to learn about CKD.

When multiple responses were allowed to the question regarding the opportunity to learn about CKD, “TV” was the most common (32.3%), followed by “newspapers” (15.3%), “from doctors or nurses” (6.4%), “magazines” (5.6%) and “from acquaintances” (5.6%), and “Internet” (2.7%) (Figure 2).

### 3.3. Understanding of the Diagnosis of CKD

**Figure 3:**
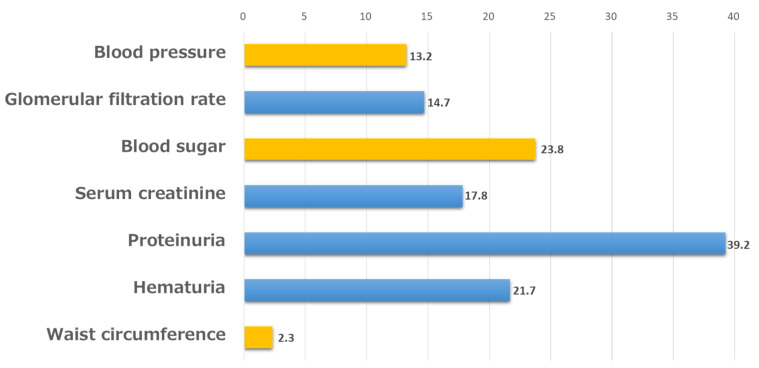
Understanding of the diagnosis of CKD.

The level of understanding of the diagnosis of CKD was summarized by multiple responses: “proteinuria”, “hematuria”, and “GFR” accounted for 39.2%, 21.7%, and 14.7% of the responses, respectively. Conversely, 23.8%, 13.2%, and 2.3% selected “blood glucose level”, “blood pressure”, and “abdominal circumference”, respectively (Figure 3). The non-response rate was 21%.

### 3.4. Survey by Age Group on Knowledge of CKD

**Figure 4:**
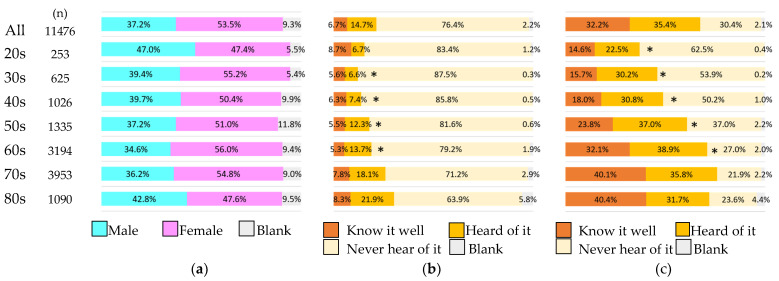
Participants and perception of CKD in a general population by age group. (**a**) Age distribution and sex of participants; (**b**) perception of “CKD” by age group; (**c**) perception of “chronic kidney disease” by age group. * *p* < 0.05 vs. 70s for “Know it well”.

Of the 15,012 respondents, we analyzed 11,476 respondents whose age and response results were available. Survey by age group showed that the older the respondents were, the higher their knowledge of both “chronic kidney disease” and “CKD” tended to be (Figure 4).

**Figure 5:**
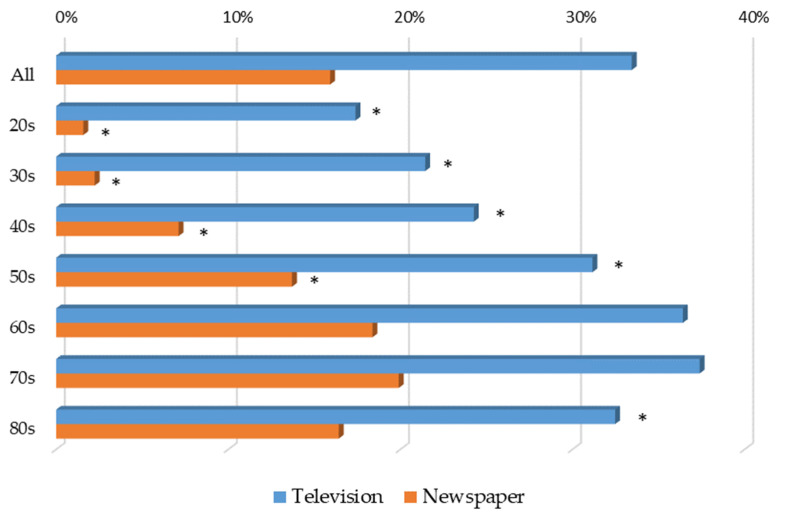
Opportunity to learn about CKD from television and newspapers by age group. * *p* < 0.05 vs. 70s for each item.

In terms of opportunities to learn about “chronic kidney disease” and “CKD”, the older the respondents were, the more they tended to use TV and newspapers (Figure 5).

**Figure 6:**
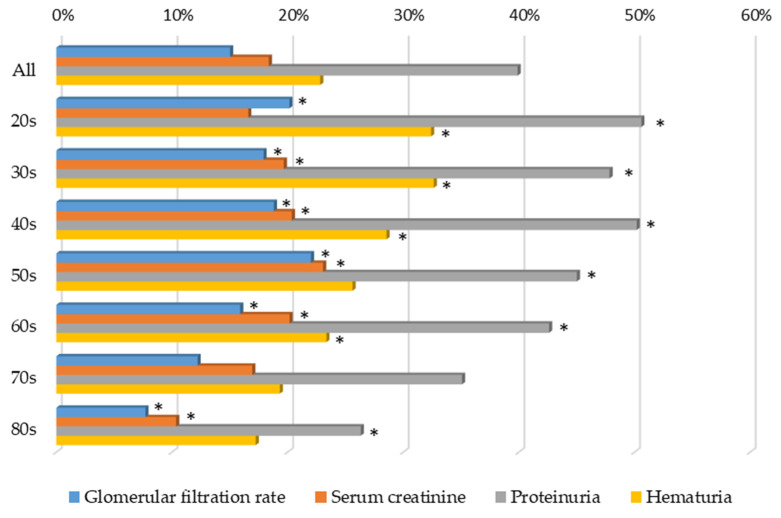
Understanding of the diagnosis of CKD, showing the percentage of correct answers by age group. * *p* < 0.05 vs. 70s for each item.

**Figure 7:**
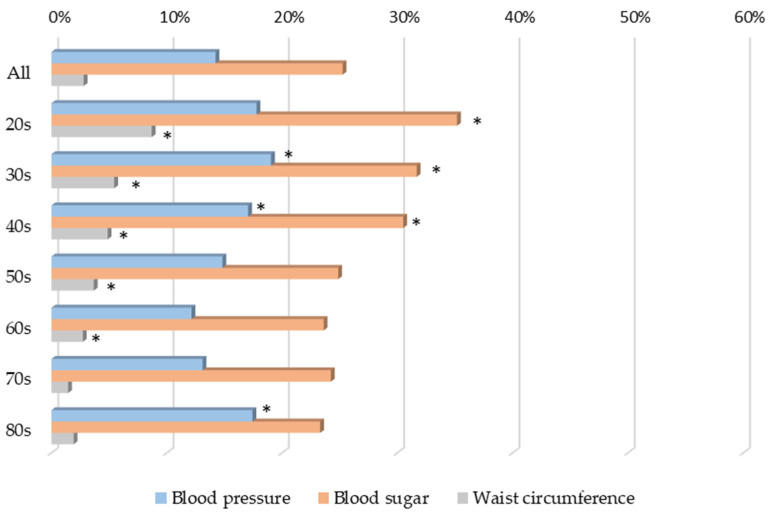
Understanding of the diagnosis of CKD, showing the percentage of incorrect answers by age group. * *p* <0.05 vs. 70s for each item.

However, the older the respondents were, the lower the percentage of correct answers (Figure 6) and the lower the percentage of incorrect answers (Figure 7) in terms of their understanding of the diagnosis of “chronic kidney disease” and “CKD”.

## 4. Discussion

In a relatively short period of time, we conducted a survey on the knowledge of CKD in 58 out of 179 cities or towns in Hokkaido prefecture and received the cooperation of a very large number of respondents. The total number of respondents was approximately 15,000, reflecting the good cooperation between medical care and the government in Hokkaido.

First, the survey revealed that the level of knowledge of chronic kidney disease (CKD) in FY2019 was insufficient: less than 50% of the respondents answered “know it well” about CKD. In particular, knowledge of “CKD” was low, at 19% for both “know it well” and “heard of it” combined. Conversely, approximately two-thirds of the respondents had at least heard of “chronic kidney disease” when “know it well” and “have heard of it” were combined, indicating that the knowledge of “chronic kidney disease” itself is increasing. However, about one-third of the remaining respondents had never heard of “chronic kidney disease” or “CKD”, suggesting the need for further educational activities. Next, we found that there was a large difference between the level of recognition of “chronic kidney disease” and “CKD”. The reason for this difference in knowledge may be that the term “CKD” is not obviously a word for kidney disease at first glance, making it difficult for the concept of “chronic kidney disease” to be established as “CKD”. In future knowledge-raising activities, it is important to determine whether it is better to promote “chronic kidney disease” first or to link “CKD” to kidney disease and promote it simultaneously and to promote knowledge-raising activities in a way that is easy for recipients to understand.

The level of understanding of the diagnosis of CKD was low as follows: “proteinuria”, “hematuria”, and “GFR” accounted for 39.2%, 21.7%, and 14.7% of the responses, respectively, suggesting that we need to further educate the general population on how to diagnose CKD. In addition, 23.8%, 13.2%, and 2.3% selected “blood glucose level”, “blood pressure”, and “abdominal circumference”, respectively, but those factors are exacerbators of CKD. Similar to our study, lower identification of the diagnosis of CKD was also reported from Hong Kong [13], Saudi Arabia [14], and USA [15]. Therefore, we also need to educate the public about the knowledge of CKD and self-management [10].

Television was the most common means of obtaining knowledge and information on CKD, followed by newspapers. In addition, hospitals and medical facilities seemed to be effective in raising CKD knowledge through flyers, posters, and direct patient guidance. However, from the general public’s perspective, there are few opportunities to obtain knowledge and information from physicians and nurses, and opportunities to learn about CKD from the Internet are limited. The reason for this is that people who go to hospitals and medical facilities have clear reasons for doing so and people search the Internet only when they are interested in chronic kidney disease (CKD), as active information gathering. In contrast, television is an easy way to obtain passive information and, thus, it plays a central role in the collection of information on medical care. Even today, when we are inundated with a variety of information, active information gathering may not be the first choice for the purpose of raising knowledge. In the future, the means of raising public knowledge should be changed in accordance with changes in society; however, at present, it is important to actively utilize mass media, such as television and newspapers, for public knowledge. Depending on the focus age group, the method of knowledge raising should be considered.

The knowledge of “chronic kidney disease” and “CKD” is not yet complete and the level of understanding of diagnosis of CKD was not high, but proteinuria was the most important item for diagnosis, at approximately 40%. Hematuria, serum creatinine level, and eGFR were the most important diagnostic items, at approximately 20%. We expect that the percentage of correct responses will increase as knowledge of CKD increases. Conversely, some respondents selected items related to metabolic syndrome, such as blood pressure, blood glucose level, and abdominal circumference. Although the high level of knowledge of metabolic syndrome can be seen in the results, it is necessary for the general public to have a correct understanding of both CKD and metabolic syndrome.

There are several limitations of this study. This survey was conducted only among those who came to the facilities cooperating with this survey for specific health checkups, which is a group with relatively high health consciousness. Since some of the respondents did not receive specific health checkups every year, the results may not accurately reflect the level of knowledge of “chronic kidney disease” and “CKD” among the general population in Hokkaido. In addition, the answers to the questions regarding the level of knowledge may lack accuracy, since no clear criteria were set and the answers were left to the judgment of individual respondents.

## 5. Conclusions

Knowledge of “chronic kidney disease” and “CKD” in Hokkaido was still below 50% as of 2019. It is possible that “chronic kidney disease” and “CKD” are not recognized as referring to the same disease and that measures are needed to reconcile this in future CKD knowledge-raising activities. These activities should be continued, since they are needed to increase the knowledge of “chronic kidney disease” and “CKD” among the general population and to raise interest in the disease itself, in order to prevent the worsening of undiagnosed or untreated kidney disease.

## Data Availability

The datasets analyzed during the current study are not publicly available, because the ethics committee approved this protocol under the condition of limited data availability.

## Supplementary Materials

The following supporting information can be downloaded at: https://www.mdpi.com/article/10.3390/jpm12111837/s1. Supplementary Table S1. Questionnaire about knowledge of chronic kidney disease (CKD).
