# Supplementary material for: Knowledge of Chronic Kidney Disease among the General Population: A Questionnaire Survey in Hokkaido Prefecture, Japan
Source: J Pers Med. 2022 Nov 3;12(11):1837. doi: 10.3390/jpm12111837 (PMC9698748; doi:10.3390/jpm12111837)
Supplement: Supplementary file 1 [file jpm-12-01837-s001.zip › jpm-1934217-supplementary.pdf]

Supplementary Table S1. Questionnaire about knowledge of chronic kidney disease (CKD).

Please complete the questionnaire about knowledge of chronic kidney disease (CKD).	
Please circle the appropriate answer to the following questions.	
(1)	What is your gender and age?
	Male, Female
	20s, 30s, 40s, 50s, 60s, 70s, 80s
(2)	Are you familiar with the term "CKD"?
	1) Have heard of it
	2) Have heard of it but do not know what it means
	3) Never heard of it
(3)	Do you know the term "chronic kidney disease"?
	1) Have heard of it
	2) Have heard of it but do not know what it means
	3) Never heard of it
If you chose "Never heard of it" for both (2) and (3), please go to (5).<br>Otherwise, please proceed to (4).	
(4)	On what occasions have you heard the term chronic kidney disease or CKD? Please choose any number of correct answers (multiple answers allowed).
	Poster/flyer, Newspaper, Television, Radio, Internet, Magazine, Public lecture, Acquaintance, Other
(5)	How is chronic kidney disease (CKD) diagnosed? Please choose any number of correct answers (multiple answers allowed).
	Blood pressure, Glomerular filtration rate (GFR), Blood glucose, Serum creatinine, Proteinuria, Hematuria, Proteinuria, Waist circumference
Thank you for your cooperation.	
